# *In silico* prediction of flavan-3-ol as a bioactive compound of *Calophyllum macrophyllum* as a potential drug against angiostrongylus eosinophilic meningitis

**DOI:** 10.14202/vetworld.2022.1305-1313

**Published:** 2022-05-25

**Authors:** Muhammad Hambal, Frengki Frengki, Wahyu Eka Sari, Henni Vanda

**Affiliations:** 1 Department of Parasitology, Faculty of Veterinary Medicine, Universitas Syiah Kuala, Banda Aceh, Indonesia; 2 Department of Pharmacology, Faculty of Veterinary Medicine, Universitas Syiah Kuala, Banda Aceh, Indonesia; 3 Department of Biochemistry, Faculty of Veterinary Medicine, Universitas Syiah Kuala, Banda Aceh, Indonesia

**Keywords:** *Angiostrongylus cantonensis*, Flavan-3-ol, molecular docking, pharmacophore

## Abstract

**Background and Aim:**

Angiostrongylus eosinophilic meningitis is caused by larvae of the rat lungworm *Angiostrongylus cantonensis*. It manifests as meningitis, radiculitis, cranial nerve abnormalities, and encephalitis, which can be fatal. A flavan-3-ol compound isolated from the bark of *Calophyllum macrophyllum* Scheff. has several medicinal properties, including antioxidant, anti-inflammatory, antidiabetic, and antibacterial activities. This compound is stronger than other types of flavan-3-ols such as catechin. This study aimed to identify the hydroxylation pattern of this flavan-3-ol compound and evaluated its potential as an anti-meningitis drug, using an *in silico* approach through pharmacophore and molecular docking methods.

**Materials and Methods:**

Pharmacokinetic and toxicological data were analyzed and supported by the server http://www.swissadme.ch/index.php and https://tox-new.charite.de/protox_II/index.php. The hydroxylation pattern of the flavan-3-ol compound was identified using shear reagents (MeOH, NaOH, NaOAc, HCl, and AlCl_3_). The CviR receptor (pdb id.3QP5) was used in the *in silico* approach, and seven ligands were downloaded from PubChem in “SMILES” format.

**Results:**

The spectroscopic analysis conducted using the shear reagents confirmed that the flavan-3-ol compound has a “*p-diOH*” pattern on the cinnamoyl ring. Pharmacophore analysis revealed this compound “*hit*” with pharmacophore features, and molecular docking analysis showed that this compound has a strong affinity with both receptors.

**Conclusion:**

The flavan-3-ol compound is a potential drug candidate for meningitis caused by pathogenic bacteria and the worm *A. cantonensis*. This result was supported by the pharmacokinetic profile, which had a very low toxicity level to the host. However, further investigation is required to confirm the data *in vitro* and *in vivo*.

## Introduction

Indonesia is rich in forest and marine natural resources, including herbs used as sources of medicines. One plant that is a potential source of bioactive chemical compounds is *Calophyllum macrophyllum*, belonging to the genus *Calophyllum*. This plant grows in Sumatra, parts of Java, Borneo, and Papua [1]. Several authors reported secondary metabolites obtained from this plant, such as coumarins, xanthones, flavonoids, biflavonoids, triterpenoids, organic acids, and benzophenone [2-5]. Biflavonoid compounds such as amentoflavone and morelloflavone were extracted for the first time by Gunatilaka *et al*. [6] from *C. calaba* that has antioxidant activity. Iinuma *et al*. [7] also obtained epicatechin from *C. inophyllum*. They also showed that this compound has antioxidant activity. Xanthones such as (+) calanolide A obtained from *C. lanigerum* were reported to be active against the human immunodeficiency virus. Other xanthone compounds possess antiplatelet, antibacterial, and antiparasitic activity against *Plasmodium falciparum* [8,9]. Our research group successfully isolated an active compound from the type *C. macrophyllum* Scheff. Phytochemical screening and spectroscopic analysis demonstrated that this compound was one of the flavan-3-ol types of the flavonoid group. Xie *et al*. [10] and Govindappa *et al*. [11] reported the antibacterial activity of flavan-3-ol compounds. The epigallocatechin-derived activity of gallate is effective against more than 17 strains of *Staphylococcus* spp., *Streptococcus pneumoniae*, *Neisseria meningitidis*, and *Klebsiella pneumoniae* through a series of *in vitro* tests [12]. These organisms are better known as pathogenic bacteria that cause meningitis.

Meningitis is an infectious and inflammatory disease of the membranes covering the brain and spinal cord with observable symptoms such as headache, fever, and stiff neck. This disease is caused by viral, fungal, and parasitic infections such as *Angiostrongylus cantonensis* [13]. *A. cantonensis*, also known as rat lung worm, can cause disease angiostrongylus eosinophilic meningitis (AEM) that infects humans through ingestion of food contaminated with larvae such as snails, raw fish, watercress, and any other raw or undercooked food [14]. Considering that the target site of *A. cantonensis* is the brain membrane, a series of studies have been conducted to find bioactive compounds against AEM [15-17].

It is important to continuously improve the discovery and development of flavonoid compounds as drugs. Flavonoid compounds are highly abundant in nature and have extensive biological activities, including treating meningitis caused by pathogenic bacteria and nematodes such as *A. cantonensis*. Several studies reported the effectiveness of flavonoid compounds against worm parasites. The anthelmintic property of quercetin, one of the flavonoid compounds against gastrointestinal nematodes, has been convincingly reported [18]. The administration of tannin and flavonoid obtained from young mango, *Mangifera indica* L., at a dose of 100 mg/mL eliminated 100% of *Strongyloides stercoralis* larvae [19]. Paul *et al*. [20] reported that flavonoids, alkaloids, and saponins of *Piper sylvaticum* Roxb. exhibited significant anthelmintic activity on the aquarium worm *Tubifex tubifex*. Another study reported that flavonoid compounds, including naringenin, flavone, hesperetin, rutin, naringin, and chrysin, eliminated *Brugia malayi* in infected rodents [21]. However, there is inadequate information on the effectiveness of flavonoid compounds against meningitis caused by *A. cantonensis*.

Molecular analytical software for determining the relationship between several nematode species using phylogenetic techniques can be the first step in predicting the effectiveness of nematotoxicity on other nematodes. Moreover, the use of pharmacophore, quantitative structure-activity relationship, and molecular docking as a virtual screening technique can help evaluate an active compound’s suitability to treat specific nematode infections.

This study aimed to investigate the medicinal property of the hydroxylation pattern of flavan-3-ol as an anthelmintic and larvicide against *A. cantonensis* *in silico* through pharmacophore and molecular docking methods followed by confirmation by pharmacokinetic data analysis.

## Materials and Methods

### Ethical approval

Since the study was carried out using computer simulation, no ethical clearance was needed.

### Data source

The sample used in this study was the flavan-3-ol compound obtained from *C. macrophyllum* Scheff, which has been identified spectroscopically [22]. The hydroxylation pattern of the cinnamoyl ring of the sample was identified by adding shear reagents using an ultraviolet (UV)–visible spectroscopy spectrophotometer (Hitachi U 2000, Japan). The shear reagents were MeOH, NaOH, NaOAc, HCl, and AlCl_3_.

To perform the *in silico* test, the samples of flavan-3-ol were used in 3D structure. The 3D structures of naringenin, catechin, quercetin, hesperetin, and chrysin were the ligands for pharmacophore features. The 3D structure of albendazole was used as the molecular docking control ligand. The ligands were downloaded from PubChem in “SMILES” format (Table-1).

**Table 1:** Control ligands downloaded from PubChem through “SMILES” format.

Compounds	PubChem ID	“SMILES FORM”
Naringenin	439246	C1C (OC2=CC(=CC(=C2C1=O) O) O) C3=CC=C (C=C3) O
Catechin	1203	C1C (C(OC2=CC(=CC(=C21) O) O) C3=CC(=C (C=C3) O) O) O
Quercetin	5280343	C1=CC(=C (C=C1C2=C (C(=O) C3=C (C=C (C=C3O2) O) O) O) O) O
Hesperetin	72281	COC1=C (C=C (C=C1) C2CC(=O) C3=C (C=C (C=C3O2) O) O) O
Chrysin	5281607	C1=CC=C (C=C1) C2=CC(=O) C3=C (C=C (C=C3O2) O) O
Albendazole	2082	CCCSC1=CC2=C (C=C1) N=C (N2) NC(=O) OC
Rutin	153184	CC1C (C(C (C(O1) OCC2C (C(C (C(O2) OC3=C (OC4=CC(=CC(=C4C3=O) O) O) C5=CC(=C (C=C5) O) O) O) O) O) O) O) O
Flavan-3-ol	-	OC1CC2=C (OC1C1=C (O) C=CC (O)=C1) C=C (O) C=C2O

The receptors were downloaded from www.rscb.org. CviR (PDB id: 3QP5) representing pathogenic bacteria [23] and AcGal-1 (PDB id: 1W6O) representing *A. cantonensis* [24].

### Experimental design

This study was conducted in several stages. First, the hydroxylation pattern of benzoyl ring and cinnamoyl flavan-3-ol was determined using shear reagents, including MeOH, NaOH, NaOAc, HCl, and AlCl_3_. The second stage was predicting the pharmacokinetics and toxicity of all ligands. The pharmacokinetics and toxicity profiles of all the tested ligands and control ligands (naringenin, catechin, quercetin, hesperetin, chrysin, albendazole, and rutin) were searched using http://www.swissadme.ch/index.php and https://tox-new.charite.de/protox_II/index.php. Based on these data, the levels of adsorption, excretion, metabolism, and toxicity of the tested ligands compared to those of the control ligands were analyzed.

The third stage was creating and optimizing the 3D structure of all ligands. The “SMILES” structure of all ligands downloaded from PubChem was converted into the 3D format using the “MOE builder” tool and saved in.mdb format. Moreover, the optimization process minimized energy to produce the most optimum ligand geometry structure. In the fourth stage, the tested ligands were analyzed based on the “pharmacophore” technique. All ligands that formed pharmacophore features were aligned using the “flexible alignment” technique to obtain a combination of formations for 3D pharmacophore development, followed by determination of the “query pharmacophore” to obtain “pharmacophore features.” Based on the “pharmacophore features,” we assessed for the presence or absence of “hit” on the tested ligands.

The final stage was analyzing the tested ligands based on the “molecular docking” technique. The ligand and receptor geometry were optimized through protonation and addition of hydrogen atoms using the 3D protonate function. Partial charge adjustment was performed using *partial charge*, energy minimization was performed with *force field* MMF94x, *gas phase* was used for solvation, and 0.001 kcal/mol gradient RMS was determined using the *default* and *file output* in.*moe* format.

The docking process began with determining the binding site based on amino acid residues that are docking targets. The “site finder” tool in MOE also guided the search for site bindings. Then, the docking was performed through the “Simulation-Dock” tool using the “triangle matcher” placement method with 1000× rounds and the “London dG” scoring function by displaying the 30 most suitable data. Furthermore, from the 30 most suitable data displayed, data refinement used a *refinement force field* with a population repetition size configuration of 1000 according to the MOE default. The display of the results of the entire docking process indicated one best result. The remaining parameters corresponded to the MOE defaults, and the output file was in.mdb format. The bonding free energy of the docking results could be observed in the output in.mdb format. Residual contact and hydrogen bonding in the best docked ligand-enzyme complex were identified and analyzed in three-dimensional media using the LigPlot software MOE and then visualized in the *ligand interaction* program.

## Results and Discussion

**Figure-1:**
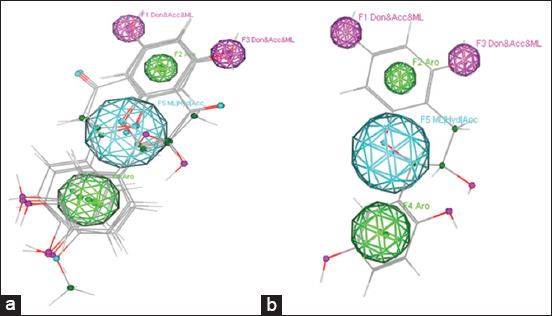
(a) Pharmacophore features constructed from naringenin, catechin, quercetin, hesperetin, and chrysin ligands. (b) pharmacophore features of flavan-3-ol ligands.

This study aimed to determine the hydroxylation pattern of the flavan-3-ol compound and identifying its potential as a meningitis drug candidate through molecular docking methods supported by analysis of pharmacokinetic data. The results of structure analysis of flavan-3-ol from UV absorbance are presented in Table-2. Results of flavan-3-ol analysis based on the “Pharmacophore” technique are shown in Figure-1. Ligand analysis based on “molecular docking” technique is listed in Table-3. Pharmacokinetic profile and ligand toxicity is shown in Table-4.

**Table 2:** Maximum absorption of UV spectrum using a shear reagent.

Shear reagent	Maximum absorbance (1_max_ [nm])	

	Band I	Band II
MeOH+NaOH	289,5	219,5
MeOH+NaOAc	280,5	223
MeOH+AlCl_3_	280,5	218,5
MeOH+AlCl_3_+HCl	280	218

**Table 3:** Ligand docking results against CviR receptors and AcGa-1 receptors.

Compounds	Receptor of CviR				Receptor of AcGal-1			
	
	Docking value (kkal/mol)	H-Bond	Distance bond (Å)	Score	Docking value (kkal/mol)	H-Bond	Distance bond (Å)	Score
Flavan-3-ol	−13.9877	Tyr 88 Tyr 88 Ser 155 Ser 155	2.10 H-Don 2.10 H-Acc 2.62 H-Don 2.62 H-Acc	98.7% 98.7% 94.0% 94.0%	−11.9881	His 2052	3.07 H-Don 3.07 H-Acc	19.6% 19.6%
Naringenin	−8.8640	Asn 77 Tyr 80	2.75 H-Acc 2.71 H-Acc	62.6% 94.2%	−10.3499	Asn 2044 Hip 2046	2.91 H-Don 2.79 H-Acc	31.7% 71.0%
Catechin	−13.1952	Tyr 80 Tyr 80 Tyr 88	2.08 H-Don 2.08 H-Acc 2.94 H-Acc	45.9% 45.9% 41.6%	−12.6204	Arg 2048 Asn 2061	2.50 H-Acc 2.97 H-Acc	17.6% 11.7%
Quercetin	−13.3896	Met 135 Ser 155 Ser 155 Ser 155	3.58 H-Don 2.75 H-Don 2.75 H-Acc 2.46 H-Acc	21.7% 58.9% 58.9% 88.9%	−12.3845	Asn 2046	2.77 H-Acc	37.3%
Hesperetin	−12.7451	Tyr 80 Tyr 80 Tyr 88 Ser 155	2.07 H-Don 2.07 H-Acc 2.36 H-Acc 2.45 H-Acc	57.0% 57.0% 100.0% 27.1%	−12.2630	Ser 2029 Hip 2044 Asn 2046	2.33 H-Acc 3.15 H-Acc 2.19 H-Acc	98.3% 10.3% 33.7%
Chrysin	−12.5559	Ser 155	2.62 H-Acc	41.4%	−10.0674	No	No	No
Rutin					−13.8851	His 2052 Gly 2069 Gly 2069 Arg 2048 Asn 2061	2.43 H-Don 3.83 H-Don 3.21 H-Don 2.78 H-Acc 2.68 H-Acc	72.3% 15.1% 52.1% 28.3% 14.2%
Albendazole	−11.4576	Tyr 80 Tyr 80 Tyr 80 Tyr 88 Tyr 88	2.97 H-Don 2.97 H-Acc 2.59 H-Acc 2.22 H-Don 2.22 H-Acc	34.7% 34.7% 25.3% 92.3% 92.3%	−9.3919	Asn 2046 Hip 2044	2.67 H-Don 2.79 H-Acc	15.1% 15.9%
Chlorolactone (native ligan of CviR)	−12.0372	Asp 97 Tyr 80 Trp 84	1.59 H-Don 2.45 H-Acc 3.22 H-Acc	72.9% 34.7% 12.4%				
Lactose (native ligand of AcGal-1)					−12.2641	Asn 2046 Gly 2069 Gly 2069 Hip 2044 Arg 2048 Arg 2048 Asn 2061 Asn 2061	1.90 H-Don 3.52 H-Don 2.95 H-Don 2.78 H-Acc 2.65 H-Acc 2.25 H-Acc 3.35 H-Acc 1.68 H-Acc	91.8% 38.9% 36.6% 77.3% 29.1% 44.0% 14.4% 38.3%

**Table 4:** Pharmacokinetic profile, toxicity level, and Lipinski criteria for each ligand.

Parameter	Ligand							

	Fv	Nr	Ct	Qr	Hp	Cs	Rt	Az
Prediction of adsorption parameter and bioavailability								
BBB	No	No	No	No	No	Yes	No	No
Human intestinal absorption	High	High	High	High	High	High	Low	High
P-glycoprotein substrate	Yes	Yes	Yes	No	Yes	No	Yes	No
Bioavailability score	0.55	0.55	0.55	0.55	0.55	0.55	0.17	0.55
LogP	1.47	2.51	1.55	1.99	2.24	2.27	2.43	1.69
TPSA (Å)	110.38	86.99	110.38	131.36	96.22	70.67	269.43	92.31
Prediction of metabolism parameter								
CYP450 1A2 inhibitor	No	Yes	No	Yes	Yes	Yes	No	Yes
CYP450 2C9 inhibitor	No	No	No	No	No	No	No	No
CYP450 2D6 inhibitor	No	No	No	Yes	No	Yes	No	No
CYP450 2C19 inhibitor	No	No	No	No	No	No	No	No
CYP450 3A4 inhibitor	No	Yes	No	Yes	Yes	Yes	No	No
Prediction of toxicity level								
Prediction of LD_50_	2.5/kg	2 g/kg	10 g/kg	0.153 g/kg	2 g/kg	3.9 g/kg	5 g/kg	0.97 g/kg
Toxicity Level	V	IV	VI	III	IV	V	V	IV
Lipinski Law								
Molecular weight (g/mol)	290.27	272.25	290.27	302.24	302.28	254.24	610.52	265.33
H-Bond acceptors	6	5	6	7	6	4	16	3
H-Bond donors	5	3	5	5	3	2	10	2
LogP	1.55	1.75	1.55	1.99	2.24	2.27	2.43	1.69
	Yes	Yes	Yes	Yes	Yes	Yes	No	Yes

TSPA=Topological polar surface area, BBB=Blood Brain Barrier

**Figure-2:**
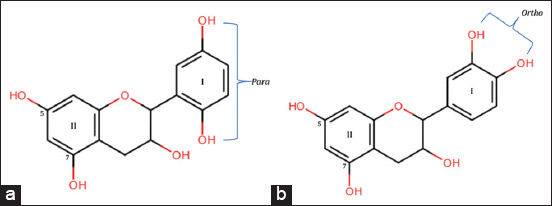
(a) 2D flavan-3-ol structure. (b) catechin structure.

The obtained flavan-3-ol compound showed an extremely similar structure to that of catechins based on previously reported spectroscopic data [22]. Catechins have a dihydroxy “*o-diOH*” pattern on the cinnamoyl ring, but we obtained a dihydroxy “*p-diOH*” pattern. The antioxidant activity was evaluated using the 2,2-diphenyl-1-picryl-hydrazyl-hydrate method, which showed that the antioxidant potential of flavan-3-ol with the dihydroxy “*p-diOH*” pattern was stronger than that of flavan-3-ol with the dihydroxy “*o-diOH*” pattern on the cinnamoyl ring. Therefore, it is necessary to confirm through the addition of shear reagent to ensure the dihydroxy pattern (Figure-2).

Adding NaOH resulted in a bathochromic shift with increasing intensity. The spectral pattern indicated the presence of a hydroxy group (OH) at position 7 on the benzene ring (band II). The slight bathochromic shift was due to the reduced acidity of the 7-hydroxy group. Meanwhile, an increase of 9.5 nm in the cinnamoyl arrangement (band I) indicated the presence of hydroxy (OH) at the 2’ position. The addition of the shear reagent NaOAc led to a bathochromic shift with increasing intensity in band II. This spectral pattern reaffirmed the presence of a hydroxyl group (OH) at position 7. Adding AlCl_3_ did not show a bathochromic shift. The spectral pattern indicated the absence of a ketone group that formed a stable complex with a hydroxy group at 3’-OH and 5’-OH positions. It also indicated the absence of an “*o-diOH*” group in the aromatic ring. Adding AlCl_3_ and HCl showed no bathochromic shift in both bands I and II. This spectral pattern confirmed the absence of ketone-hydroxy and “*o-diOH*” complexes in the aromatic ring [25]. This analysis indicated that the isolated type of flavan-3-ol had a dihydroxy “*p-diOH*” pattern on the cinnamoyl ring with the molecular structure 5, 7, 2’, 5’-tetrahydroxy flavan-3-ol.

Pharmacophore is a ligand-based drug design technique. Functional groups and unique conformations attached to biologically active compounds become references in drug design, known as pharmacophore features. In this study, five flavonoid compounds had antibacterial activity [10,26] and anthelmintic activity [18,21]. The results of the alignment of these five compounds indicated five pharmacophore features, including F1 (Don and Acc and ML), F2 (Aro), F3 (Don and Acc and ML), F4 (ML/Hyd/Acc), and F5 (Aro). The five annotation points were based on the formation of all control ligands that comprise the “pharmacophore” feature.

The pharmacophore analysis revealed the flavan-3-ol compound “hit” in all the “pharmacophore” features that included the aromatic structure of benzoyl and cinnamoyl rings, two hydroxy groups of the benzoyl ring, and the ether group (−O−). This is simply due to the structural similarity of the flavan-3-ol compound with the ligands that comprised the pharmacophore features. Hence, the flavan-3-ol compound also had antibacterial and anthelmintic properties.

In addition to the pharmacophore technique, molecular docking predicted the potential of flavan-3-ol as a meningitis drug candidate. Albendazole and chlorolactone (native ligand) were control ligands at the CviR receptor, as reported by Singh and Bhatia [27]. The CviR receptor is one of the LuxR family proteins [28]. This protein plays a key role in activating the transcription of sensing genes, including the virulence genes of various bacterial meningitis pathogens [29-31]. However, suppressing CviR activity reduces the expression of these pathogenic virulence genes. Annapoorani *et al*. [32] conducted an *in silico* analysis of the mechanism underlying the inhibition of the LuxR family receptor to explore the active compound capable of suppressing the LasR and RhlR proteins of *Pseudomonas aeruginosa*.

Docking validation used a redocking technique on the native ligand chlorolactone, and a root mean square deviation (RMSD) value of 2.3 was obtained. Kotnik *et al*. [33] mentioned that the RMSD value of 2.5 matched with the standard validation method enabling the docking process to continue for other ligands.

**Figure-3:**
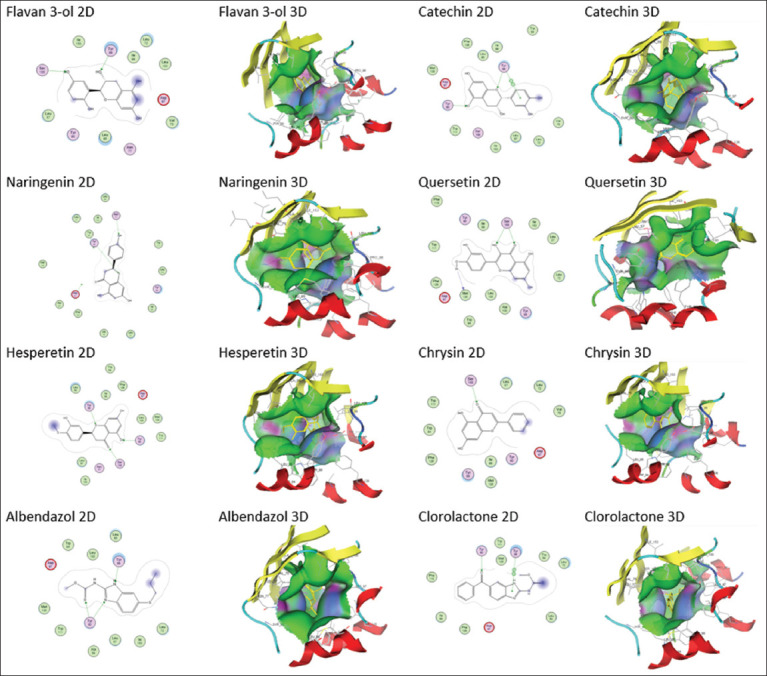
The interaction model of the test and control ligands on the CviR receptor.

The docking analysis revealed that flavan-3-ol had the strongest docking score compared with control flavonoid derivatives (naringenin, catechin, quercetin, hesperetin, and chrysin), albendazole, and even native ligands when forming complexes with the CviR receptor (Table-3). The strength of the interaction between flavan-3-ol and the CviR receptor was due to the strong dominance of the hydrogen bond with the percentage values of Tyr 80 and Ser 155 amino acid residues of 98.7% and 94%, respectively. Naringenin had the weakest affinity with two hydrogen bonds at the amino acid residues Asn 77 and Tyr 80, with percentages of 62.6% and 94.2%, respectively. The 3D observations revealed that the cinnamoyl naringenin ring tends to fold away from the inner side of the binding site (Figure-3). However, the other ligands tend to go deeper into the binding site. Nevertheless, Singh and Bhatia [274] reported that the hydrophobic binding of albendazole was also essential in the interaction with the CviR receptor. Meanwhile, flavan-3-ol had a more dominant hydrophilic side, and hence, the effectiveness of this test ligand must be confirmed *in vitro*.

**Figure-4:**
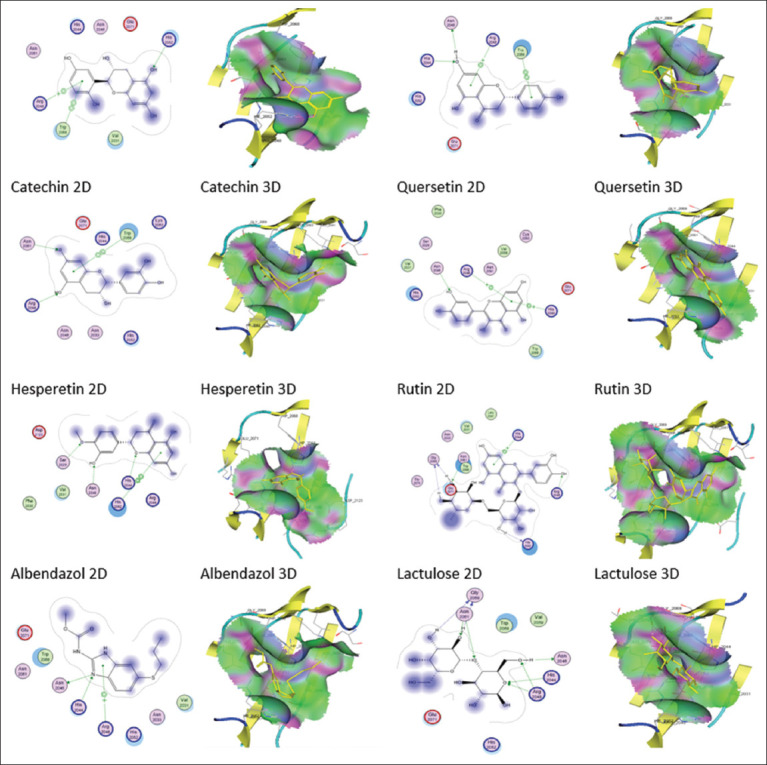
The interaction model of the test and control ligands on the AcGal-1 receptor.

The docking analysis on the AcGal-1 receptor on *A. cantonensis* indicated that this protein interacted with annexin A2 protein, a surface receptor for numerous host cells in humans, including macrophage cells (Figure-4). This interaction activated the JNK pathway which resulted in the process of apoptosis and macrophage cell necrosis [34]. This mechanism was considered the strategy by which *A. cantonensis* evades host immune response [14].

At the AcGal-1 receptor (PDB id. 1W6O), lactose was identified as a native ligand that acted as an inhibitor. There has been an increasing focus on the discovery and development of inhibitors, generally in the form of “lactose-based inhibitors” [35]. The potential of the flavan-3-ol compound and that of several other flavonoid compounds were analyzed, either in a single position or in a state bound to sugar (glycosidic bonds such as rutin). The ligand docking process with the AcGal-1 receptor was performed in the carbohydrate recognition domain region, which included His-44, Asn-46, Arg-48, His-52, Asn-61, Trp-68, Glu-71, and Arg-73 [36]. The docking results showed that all ligands had a strong potential to bind to receptors. Lactose as a native ligand control demonstrated a strong potential with a docking value of −12.2641 kcal/mol. The eight hydrogen bonds supported this affinity’s strength (Table-3). Other ligands, including flavan-3-ol, also had a strong affinity but less than that of lactose.

Quercetin derivatives (rutin) that had glycosidic bonds with sugars also exhibited the strongest affinity with the AcGal-1 receptor, with docking values reaching −13.8851 kcal/mol. This was due to the five hydrogen bonds with four bonds from the sugar. The strength of this bond was largely determined by the presence of sugar with a hydroxyl group that formed hydrogen bonds with the receptor. However, flavan-3-ol also exhibited a strong potential as an AcGal-1 inhibitor, as evidenced by the docking value of −11.9881 kcal/mol with two hydrogen bonds (Table-3).

The test ligands and control ligand compounds, except rutin, had log P<5 and topological polar surface area (TPSA) <140, so they had a fairly large bioavailability value (0.55), whereas rutin had a higher log P (2.43) and TPSA (269.43). However, its absorption and distribution ability were lower, so its bioavailability was also low (0.17) (Table-4). Lawson *et al*. [37] reported that the physicochemical properties of drug compounds varied from one another, and even a slight difference in the side chain can cause a significant change in their interaction with the receptor. Pharmacokinetics can also be observed through changes in the drug’s adsorption ability. The adsorption process of compounds is affected by several factors such as lipophilicity, hydrogen bonding, molecular size, and pKa charge. Ideally, drug compounds can penetrate cell membranes and have a strong affinity for target receptors [38]. The cell membrane tends to be lipophilic because it is rich in phospholipid content. The cytosol tends to be hydrophilic as it contains protein and water. Lipophilic properties play an essential role during the absorption and distribution phase of the drug so that lipophilicity significantly determines the bioavailability of a drug in the body. Log P and TPSA are parameters commonly used in evaluating the lipophilicity of an active substance [39].

Flavan-3-ol may be safe for ingestion with other drug combinations because it does not inhibit several cytochrome P450 metabolic enzymes. However, flavan-3-ol is also a Pgp protein substrate that acts as a “drug efflux” that immediately pumps flavan-3-ol back into the extracellular fluid, reducing the intracellular concentration of flavan-3-ol. This condition could suppress the effectiveness of flavan-3-ol and trigger resistance [40-42]. Flavan-3-ol also had a low level of toxicity (level V) with a lethal concentration 50 of >2.5 g/kg body weight, and thus, it was included in the safer category. This compound has a molecular weight of 290 g/mol with a LogP of 1.55, and the number of H-donors and H-acc is 6 and 5, respectively (Table-4). Therefore, flavan-3-ol qualifies as a good candidate drug compound according to the rules of Lipinski [42].

## Conclusion

The shear reagent analysis confirmed that flavan-3-ol had a “*p*-diOH” pattern on the cinnamoyl ring with the chemical formula 5, 7, 2’, 5’-tetrahydroxy flavan-3-ol. Based on pharmacophore analysis and molecular docking, this compound could be a candidate drug for meningitis caused by infection with pathogenic bacteria and the parasite *A. cantonensis*. Clinically, this compound exerted a better effect than albendazole and the other tested flavonoid compounds. Nonetheless, these compounds have not been tested in laboratory experiments. These results are still in the stage of confirming that the structure of *para* (p) in the cinnamoyl ring had stronger pharmacological activity than the *ortho* (o) of flavonoid compounds. Therefore, further research is required to analyze the effect of flavan-3-ol obtained from *C. macrophyllum* as an anthelmintic and antibacterial drug using *in vitro* and *in vivo* approaches.

## Authors’ Contributions

MH, HV, and FF: Contributed to the original draft, analysis, and interpretation of data. MH and WES: Conception and design of the study. HV and FF: Revised the manuscript and improved English language of the manuscript. All authors read and approved the final manuscript.
